# Research on cognitive and sociocognitive functions in patients with brain tumours: a bibliometric analysis and visualization of the scientific landscape

**DOI:** 10.1007/s10072-020-04276-x

**Published:** 2020-02-12

**Authors:** Milena Pertz, Stoyan Popkirov, Uwe Schlegel, Patrizia Thoma

**Affiliations:** 1 Department of Neurology, University Hospital Knappschaftskrankenhaus, Ruhr-University Bochum, In der Schornau 23-25, D-44892 Bochum, Germany; 2 Neuropsychological Therapy Centre (NTC)/Clinical Neuropsychology, Faculty of Psychology, Ruhr-University Bochum, Universitätsstraße 150, D-44780 Bochum, Germany

**Keywords:** Brain tumour, Cognition, Social cognition, Bibliometric analysis, VOSviewer

## Abstract

**Background:**

Many patients with brain tumours exhibit mild to severe (neuro)cognitive impairments at some point during the course of the disease. Social cognition, as an instance of higher-order cognitive functioning, specifically enables initiation and maintenance of appropriate social interactions. For individuals being confronted with the diagnosis of a brain tumour, impairment of social function represents an additional burden, since those patients deeply depend on support and empathy provided by family, friends and caregivers.

**Methods:**

The present study explores the scientific landscape on (socio)cognitive functioning in brain tumour patients by conducting a comprehensive bibliometric analysis using VOSviewer. The Web of Science Core Collection database was examined to identify relevant documents published between 1945 and 2019.

**Results:**

A total of 664 English titles on (socio)cognitive functions in patients with brain tumours was retrieved. Automated textual analysis revealed that the data available so far focus on three major topics in brain tumour patients: cognitive functions in general and in paediatric cases, as well as psychological factors and their influence on quality of life. The focus of research has gradually moved from clinical studies with cognitive functions as one of the outcome measures to investigations of interactions between cognitive functions and psychological constructs such as anxiety, depression or fatigue. Medical, neurological and neuropsychological journals, in particular neuro-oncological journals published most of the relevant articles authored by a relatively small network of well interconnected researchers in the field.

**Conclusion:**

The bibliometric analysis highlights the necessity of more research on social cognition in brain tumour patients.

## Introduction

Various cognitive subprocesses, such as attention, memory and executive functions, mediate our performance in occupational, social and everyday life and affect participation and quality of life (QoL) in patient populations, such as those affected by brain tumours [1–6]. Most patients with brain tumours exhibit (neuro)cognitive impairments at some point [7] turning cognition into an important outcome measure, even more since newer therapies have prolonged survival [8, 9]. The degree of cognitive impairment varies from mild to severe across patient populations, related to disease and treatment variables, methodological issues, duration of follow-up and population discrepancies in different studies [10–12].

In recent years, social cognition, as an instance of higher-order cognitive functioning, has sparked some interest in the neuro-oncological community. It represents an umbrella term for psychological constructs that vary in their complexity, ranging from more elementary perceptual functions, such as emotion recognition, to more elaborate ones, such as empathy, Theory of Mind and social problem-solving [13, 14]. Since initiation and maintenance of appropriate social interactions rely mainly on the ability to successfully decode mental and emotional states of other individuals, these sociocognitive abilities critically contribute to social integration, participation as well as overall mental health, wellbeing and QoL. Impairments of sociocognitive functions, due to treatment and/or the brain tumour itself, might contribute to social isolation and shrinking social networks [15–17] which is disastrous as social support is crucial to protect people in highly demanding life situations from pathological distress [18].

Both cognitive and sociocognitive functions are of particular relevance for patients with brain tumours. This is also true for those diagnosed with grade I–III gliomas according to the classification of the World Health Organization (WHO) as these typically affect young adults with an anticipated survival of many years [19]. Therefore, patients with a WHO-grade I–III glioma have to live and cope with potential impairments of (socio)cognitive functions for many years and even decades. Furthermore, in highly demanding situations, such as diagnosis and treatment of a life-threatening illness (e.g. WHO-grade IV tumours), patients rely on the support of their family members and other caregivers. However, impairment of social function may prevent those patients from interacting adequately with their environment.

Taken together, accurate assessment of functioning in (socio)cognitive domains in brain tumour patients is important for patient counselling, treatment planning, therapeutic decision-making and potential rehabilitation and reintegration programs. To explore the ways in which social cognition has been addressed in Neuro-Oncology research so far, a bibliometric analysis of the scientific landscape was performed that can identify clusters and trends in research on cognitive and sociocognitive functions in patients with brain tumours.

## Methods

### Data collection

Relevant literature was identified using the Web of Science Core Collection database as a collection of over 20,000 peer-reviewed journals published worldwide in over 250 disciplines. Search terms were defined based on which cognitive domains (e.g. attention, information speed, visual construction, execution, working memory, verbal and visual memory) appear to be most consistently affected in brain tumour patients based on previous literature [11, 19–22] as well as on which subprocesses of social cognition are relevant in highly demanding life situations like diagnosis and treatment of a brain tumour (e.g. emotion recognition, empathy, Theory of Mind, social skills and social problem-solving). The search of relevant documents of the main analysis was limited to the titles of publications in the Web of Science Core Collection database applying the following search string:

TI = ((“social cognition” OR “theory of mind” OR “mentaliz*” OR “empath*” OR “emotion recognition” OR “social problem solving” OR “social skills” OR “cognit*” OR “memory” OR “execut*” OR “attention” OR “information speed” OR “visual construction”) AND (“brain tumour*” OR “brain tumor*” OR “brain neoplasm*” OR “intracranial neoplasm*” OR “brain cancer*” OR “intracranial tumour*” OR “intracranial tumor*” OR “glioma*” OR “meningioma*” OR “primary central nervous system lymphoma*” OR “brain metastases” OR “brain metastasis”)).

Furthermore, an additional subanalysis of content explicitly focusing on brain tumour patients with good chances of medium- or long-term disease control such as meningiomas, neurinomas, low-grade gliomas and primary central nervous system lymphomas was carried out to investigate the impact of (socio)cognitive functions in patient populations that have to cope with potential impairments of those functions for many years or even decades. The search of relevant documents of the subanalysis was limited to the titles of publications in the Web of Science Core Collection database applying the following search string:

TI = ((“social cognition” OR “theory of mind” OR “mentaliz*” OR “empath*” OR “emotion recognition” OR “social problem solving” OR “social skills” OR “cognit*” OR “memory” OR “execut*” OR “attention” OR “information speed” OR “visual construction”) AND (“meningioma*” OR “neurinoma*” OR “low grade glioma*” OR “low-grade glioma*” OR “primary central nervous system lymphoma*”)).

Only articles in English were included and the search spanned a period from 1945 to the end of 2019. Since meeting abstracts tend to reflect organizational logistics rather than editorial decisions, they were excluded from the analysis of relevant sources. For all other analyses, they were included. (Meta)data of documents were imported to VOSviewer version 1.6.11, a software tool for constructing, analysing and visualizing bibliometric maps [23–25]. All further analyses and visualizations described below were conducted using VOSviewer. To limit the potential impact of irrelevant or duplicate titles, the document titles were screened for relevance and uniqueness. The search was carried out on January 2, 2020.

### Term maps

Terms were automatically extracted from the titles and abstracts of all documents in the datasets and were used to construct maps, for instance network and density visualizations, based on textual data [25]. Terms were counted in a binary fashion, meaning that each term was counted only once per item [26]. A customized “thesaurus” was used to avoid redundancy and synonyms, for instance “whole brain radiotherapy”, “wbrt” and “whole brain radiation therapy” counted as the same term. To label each identified term as a relevant source, the minimum number of occurrences of a term was set to ten in the main analysis of content. For the subanalysis, the minimum number of occurrences of a term was set to five, due to the lower number of documents for this detailed analysis. The top 60% of the terms identified according to the scores were included in the analysis as a default setting. Furthermore, all terms were manually inspected and uninformative general usage terms such as “end”, “article” or “author” were excluded.

### Co-authorship map

A visualization of co-authorship networks in the field of cognitive and sociocognitive functions in patients with brain tumours was constructed using the dataset of the main analysis by creating a map based on bibliographic data. The counting method was fractional, meaning that the weight of a link is fractionalized. For instance, if an author co-authors a document with ten other authors, each of the ten co-authorship links has a weight of 1/10 [26]. Of the total number of authors, the minimum number of documents of an author was set to five and the minimum number of citations of an author was set to one for further automatic clustering and network visualization. For each of the authors, the total strength of the co-authorship links with other authors was calculated. Again, a customized “thesaurus” was used to avoid redundancy, for instance “Correa, D. and “Correa, D. D.” counted as the same author.

### Map of sources

As mentioned before [27], the dataset was reduced by excluding meeting abstracts before performing the map of sources analysis since they could distort the network properties in favour of official journals or societies. For all other analyses, they were included. The network structure of scientific journals in the field of cognitive and sociocognitive functions in patients with brain tumours was explored by creating a map of sources within the dataset of the main analysis that visualizes the relatedness of publication sources based on the number of times they cite each other. The counting method was again fractionalized: The minimum number of documents of a source/journal was three and the minimum number of citations of a source/journal was one. The total strength of the citation links with other journals was calculated.

## Results

### Datasets

**Fig. 1:**
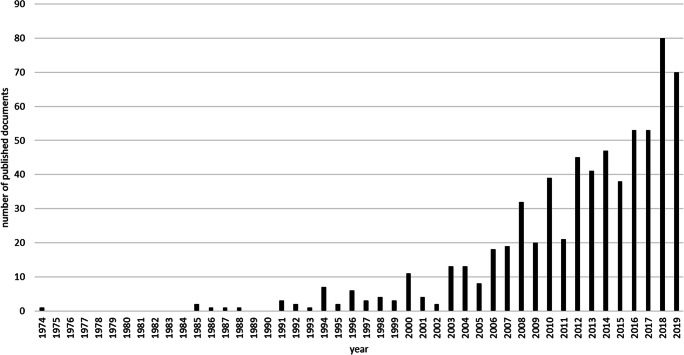
Main analysis. Number of published documents on cognitive and sociocognitive functioning in brain tumour patients per year. Visualization starts at 1974 since the first publication was detected in 1974

In the main analysis, a total of 664 documents with titles referring to cognitive or sociocognitive functions in patients with brain tumours were identified. As reported in Fig. 1, the number of published documents rises per year and shows a trend of increasing publication output. Since the first relevant publication was identified in 1974, Fig. 1 shows the time course from 1974 onwards. This might be due to the fact that computer tomography and magnetic resonance imaging evolved in the 1970s and 1980s [28] for use in clinical practice allowing for preoperative neuroimaging and brain tumour diagnosis. Therefore, the first studies concerning lesion characteristics and (neuro)cognition were published from that time onwards.

For the subanalysis, a total of 88 documents with titles referring to cognitive or sociocognitive functions in brain tumour patients, in whom long-term disease control can be achieved, were found. Since the first relevant publication was identified in 1994, Fig. 2 shows the time course from 1994 onwards. Research on (socio)cognitive functions in brain tumour patients, in whom prolonged disease control is possible, seems to be a relatively recent focus in research. This might be due to the fact that newer treatment options have prolonged survival in those patients in the last decades and factors influencing occupational and social reintegration, for instance (socio)cognitive abilities, became more important in those patient populations.

**Fig. 2:**
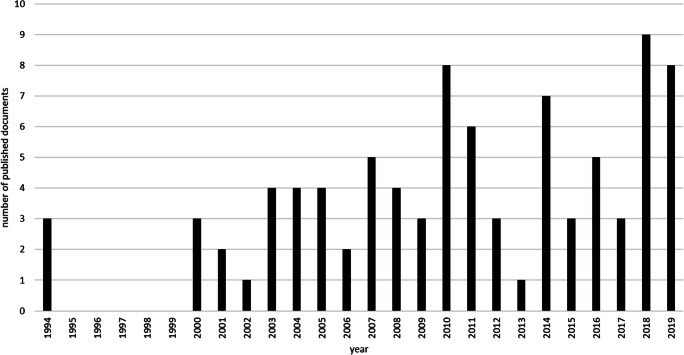
Subanalysis. Number of published documents on cognitive and sociocognitive functioning in brain tumour patients per year. Visualization starts at 1994 since the first publication was detected in 1994

### Term maps

#### Main analysis

A total number of 8412 terms was automatically identified from the titles and abstracts of all 664 documents. Of those, 181 occurred at least ten times. The top 60% of those 181 terms were selected (109 terms) and manually inspected to avoid uninformative general usage terms as described earlier (“Term maps” in methods section). The remaining 42 terms were analysed and visualized.

**Fig. 3:**
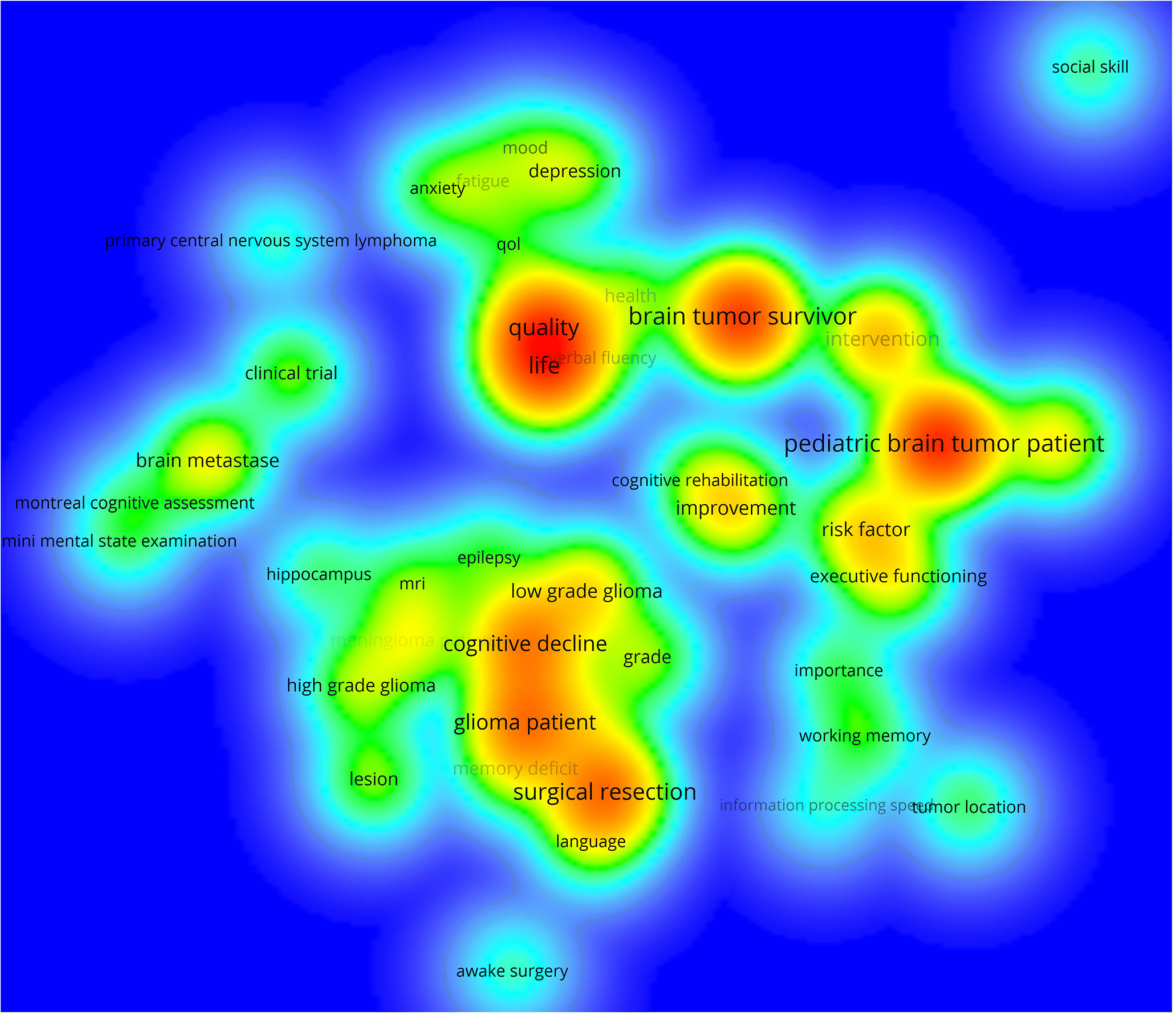
Generated term map (density visualization) of selected terms identified from the titles and abstracts of all 664 documents representing areas of intensively researched domains in the field of cognitive and sociocognitive functions in brain tumour patients. Colour intensity is scaled to the number of (binary) occurrence of terms at each specific point and the co-occurrence of the neighbouring terms

For an overview of overall trends in research, Fig. 3 shows a density visualization of the extracted terms. For each point in this visualization, the colour indicates the density of terms at that point, measured in terms of occurrence and co-occurrence. The higher the number of occurrence of terms at that point and the higher the co-occurrence of the neighbouring terms, the closer the colour intensity of the point is to red [26]. In other words, Fig. 3 presents areas of intensively researched domains in the field of (socio)cognitive functions in brain tumour patients and their interconnections.

**Fig. 4:**
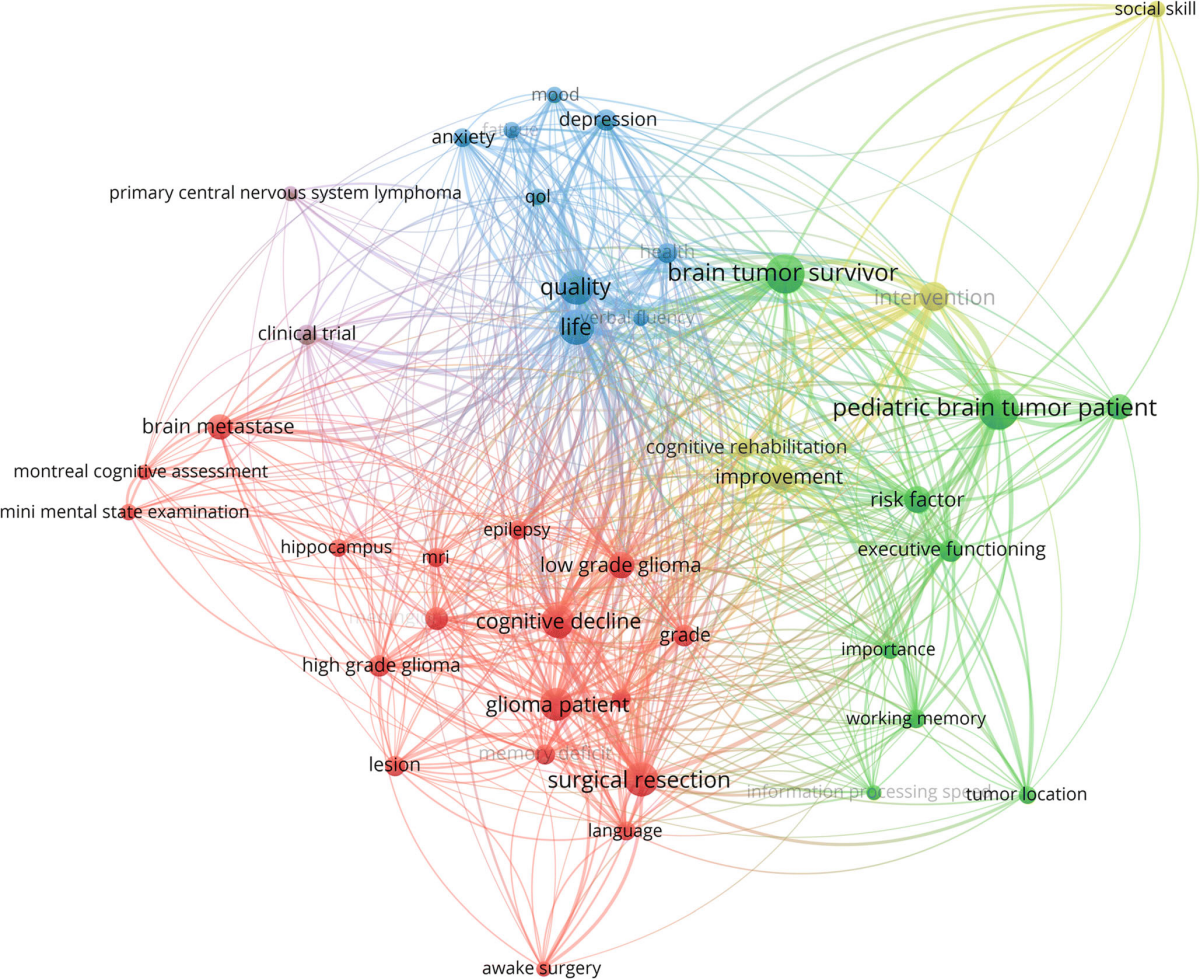
Generated term map (network visualization) of selected terms identified from the titles and abstracts of all 664 documents representing three major clusters (red, green and blue) and two minor thematic clusters (yellow and purple) based on term co-occurrence in research on cognitive and sociocognitive functions in patients with brain tumours. Circle size is scaled to the total number of (binary) occurrence of each term. Lines between terms indicate co-occurrence. Colours denote clusters based on term co-occurrence

Figure 4 shows the generated term map detailing on clusters of closely related terms in a network visualization. The larger the circle, the higher the frequency of occurrence of the specific term and the smaller the distance between two terms/circles, the higher the co-occurrence of the terms. Colours indicate clusters of closely related terms. Cluster analysis based on term co-occurrence identified three major clusters (red, green and blue) and two minor thematic clusters (yellow and purple).

**Fig. 5:**
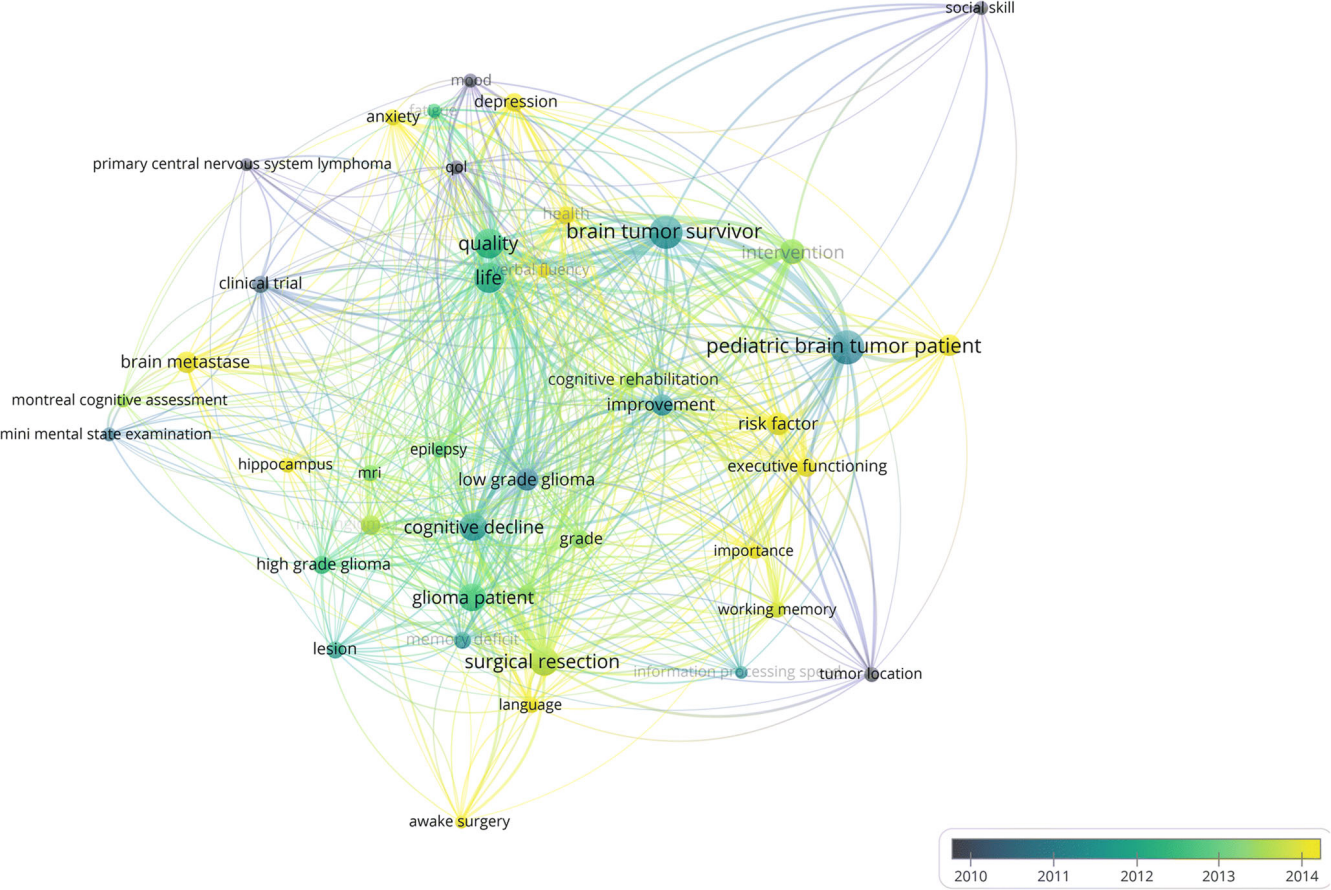
Generated term map (network visualization) of selected terms identified from the titles and abstracts of all 664 documents with chronological overlay to visualize tendencies in research on cognitive and sociocognitive functions in brain tumour patients over time. Circle size is scaled to the total number of (binary) occurrence of each term. Lines between terms indicate co-occurrence. Colours indicate average publication year of terms (see colour scale)

Figure 5 shows the same network visualization of the term map colour-coded for time (average publication year of term) to visualize tendencies in research on cognitive and sociocognitive functions in brain tumour patients over time.

#### Subanalysis

A total number of 1610 terms were automatically identified from the titles and abstracts of 88 documents. Of those, 63 occurred at least five times. The top 60% of those 63 terms were selected (38 terms) and manually inspected to avoid uninformative general usage terms as described earlier (“Term maps” in methods section). The remaining 20 terms were analysed and visualized.

**Fig. 6:**
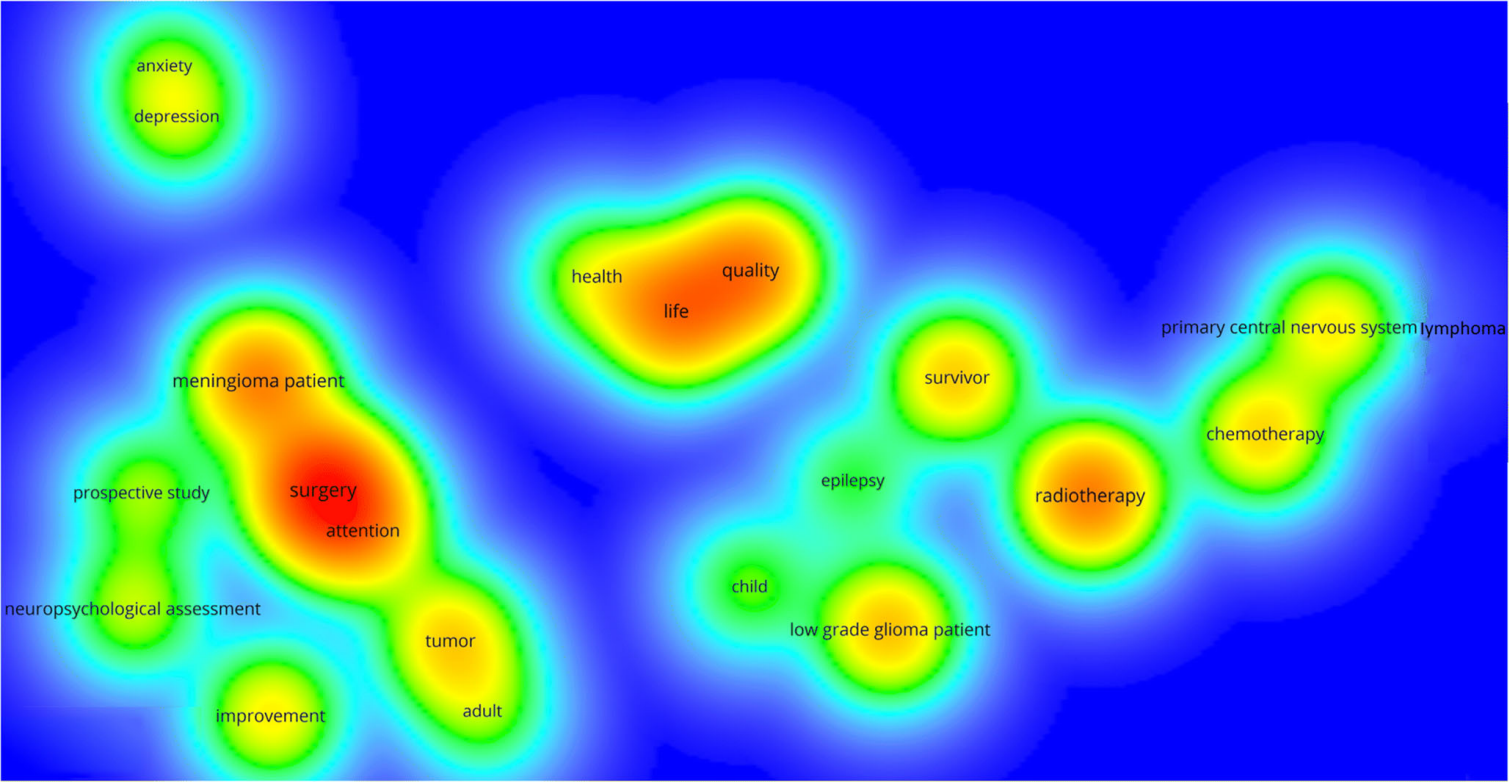
Generated term map (density visualization) of selected terms identified from the titles and abstracts of 88 documents representing areas of intensively researched domains in the subanalysis. Colour intensity is scaled to the number of (binary) occurrence of terms at each specific point and the co-occurrence of the neighbouring terms

Figure 6 shows the extracted terms in a density visualization to provide an overview of intensively researched domains in the field of cognitive and sociocognitive functions in brain tumour patients, in whom medium- or long-term disease control can be achieved.

**Fig. 7:**
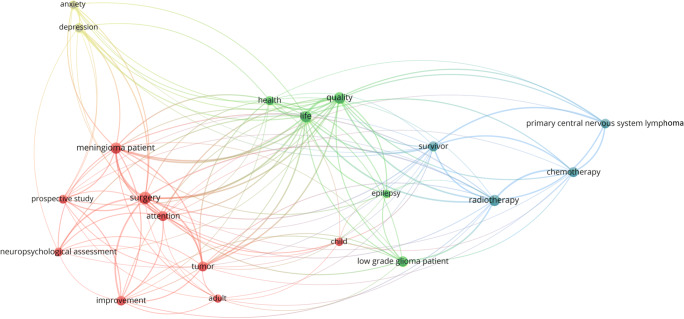
Generated term map (network visualization) of selected terms identified from the titles and abstracts of 88 documents representing three major clusters (red, green and blue) and one minor thematic cluster (yellow) based on term co-occurrence in the subanalysis. Circle size is scaled to the total number of (binary) occurrence of each term. Lines between terms indicate co-occurrence. Colours denote clusters based on term co-occurrence

Figure 7 presents the generated term map detailing on clusters of closely related terms in a network visualization. Cluster analysis based on term co-occurrence identified three major clusters (red, green and blue) and one minor thematic cluster (yellow).

**Fig. 8:**
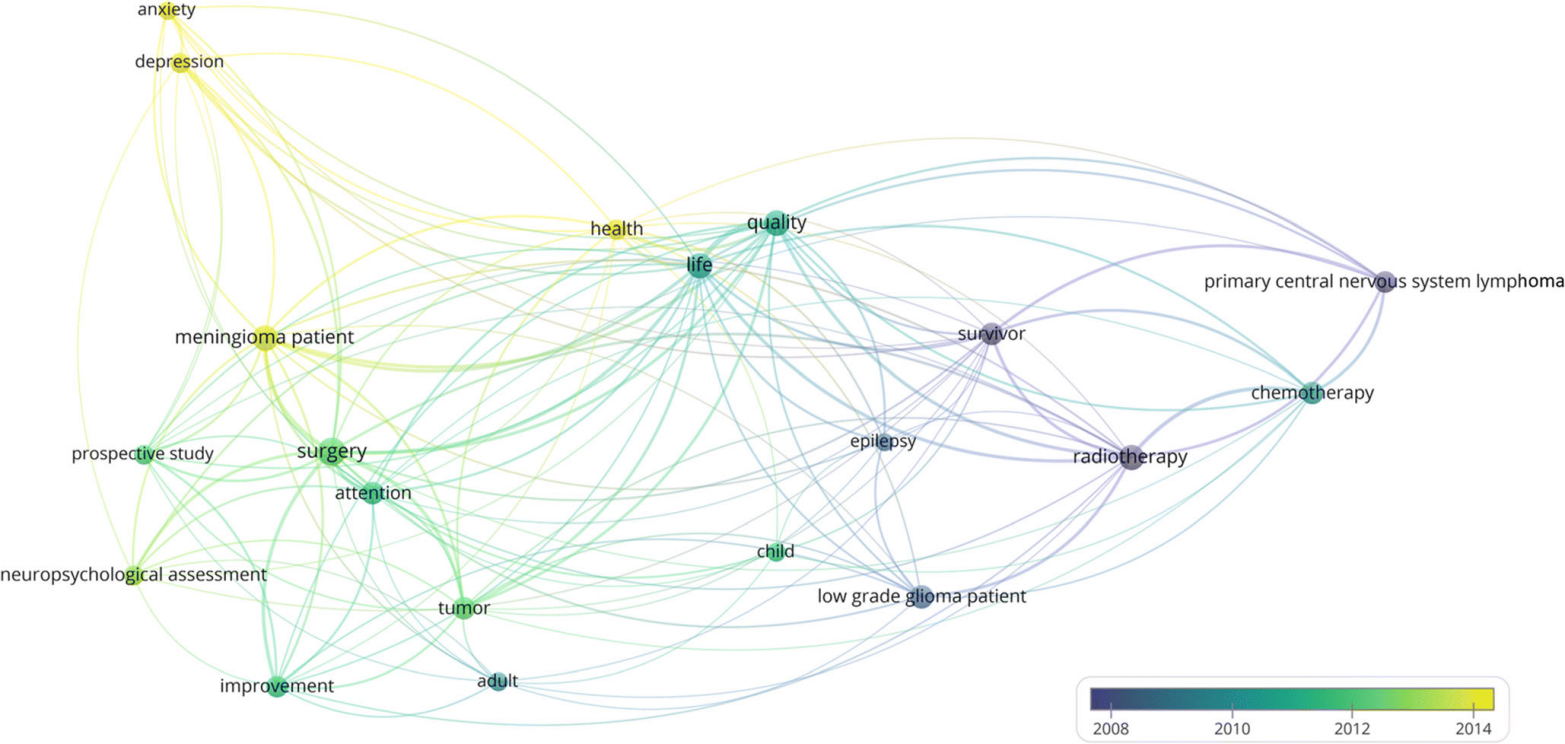
Generated term map (network visualization) of selected terms identified from the titles and abstracts of 88 documents with chronological overlay to visualize research tendencies in the subanalysis over time. Circle size is scaled to the total number of (binary) occurrence of each term. Lines between terms indicate co-occurrence. Colours indicate average publication year of terms (see colour scale)

Figure 8 represents the same network visualization of the term map colour-coded for time (average publication year of term).

### Co-authorship map

**Fig. 9:**
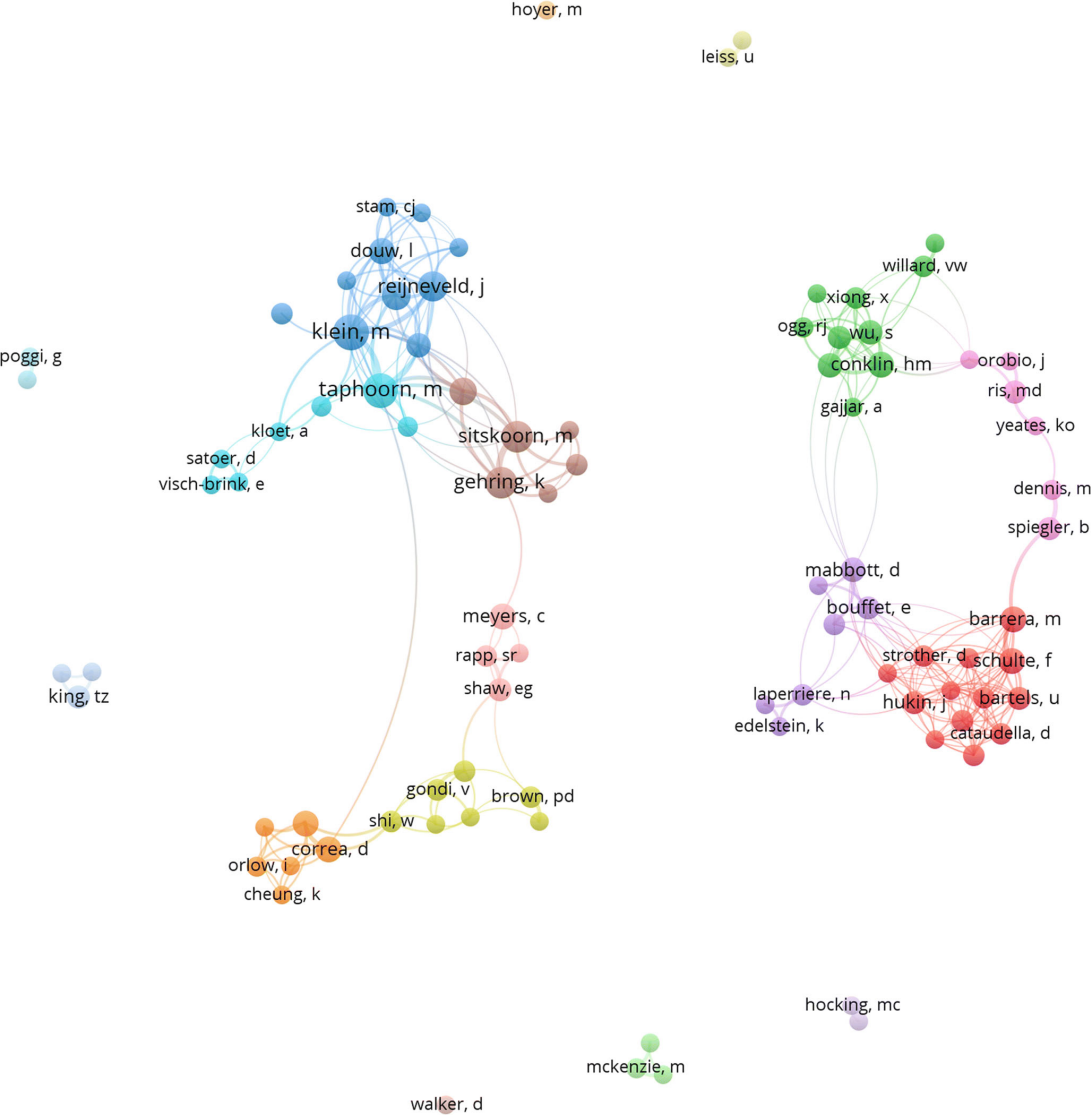
Generated co-authorship map from all 664 documents to visualize the most prolific authors and their cooperation based on the number of co-authored documents. Circle size is scaled to the number of documents published. Links represent co-authorships. Colours represent clusters based on co-authorships

A total of 2519 authors were identified in all 664 documents. Of those, 91 met the predefined thresholds of a minimum number of five documents and one citation per author. Figure 9 shows these 91 authors with automatic colour-coded clusters for cooperation based on co-authorship.

### Map of sources

This analysis was performed after excluding all documents that were classified as “Meeting abstract” or “Meeting summary” and was thus based on 378 documents.

**Fig. 10:**
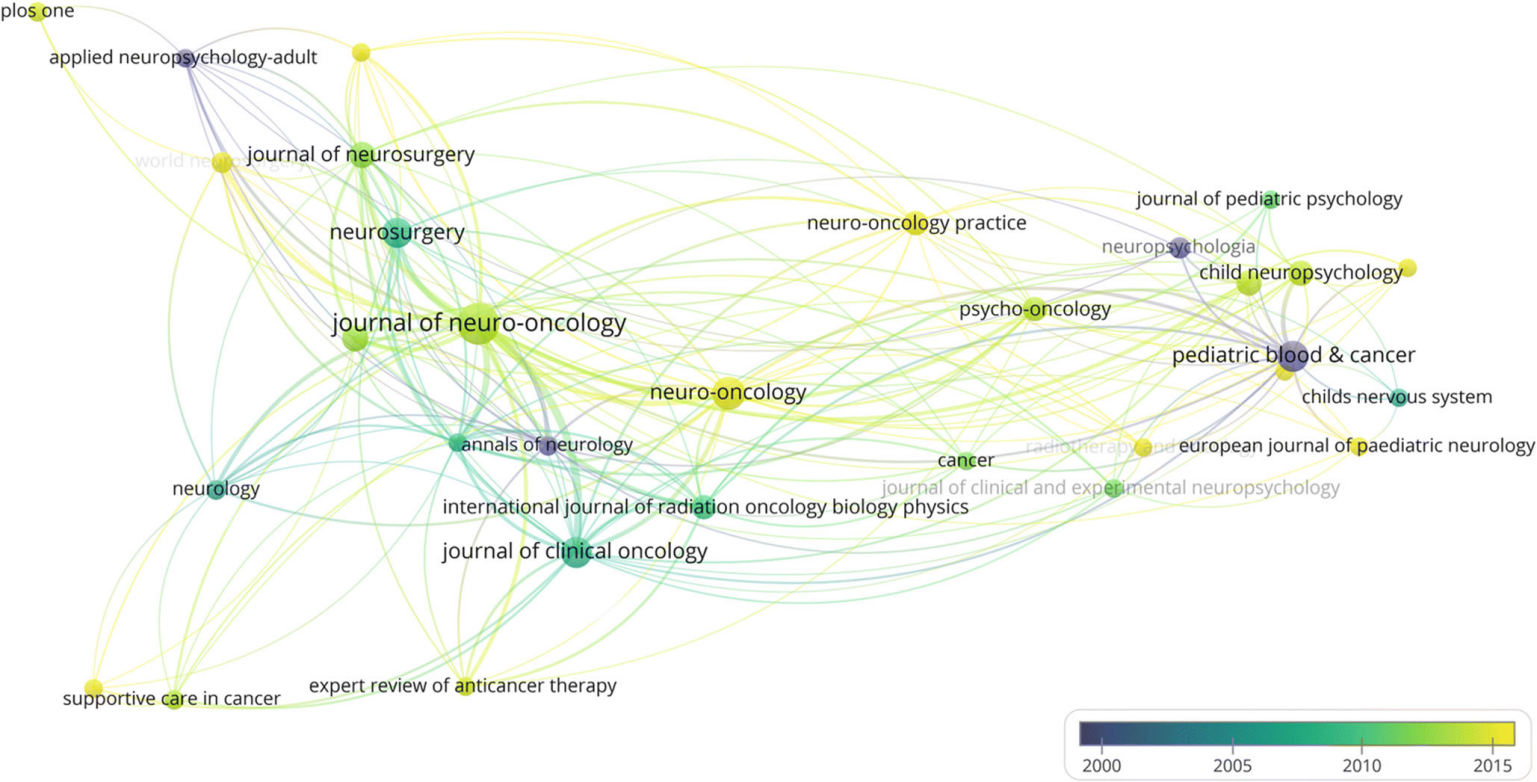
Generated map of sources from 378 documents excluding meeting abstracts and meeting summaries to visualize the most impactful sources in the field of cognitive and sociocognitive functions in brain tumour patients based on the number of times they cite each other with chronological overlay to visualize tendency in publication properties over time. Circle size is scaled to the number of published documents. Links indicate the citations between sources. Colours represent average publication year of all documents published by each source (see colour scale)

In total, 146 sources were identified and 31 met the predefined criterion of a minimum number of three documents and one citation per source. Figure 10 visualizes the relatedness of sources based on the number of times they cite each other with chronological overlay. Several major general neurology and neurosurgery journals, general medical journals but also specified journals for Neuro-Oncology and neuropsychology emerged along with journals concerning paediatric research.

## Discussion

The bibliometric analysis of 664 scientific documents addressing (socio)cognitive functions in patients with brain tumours yielded several interesting findings and illustrates that the scientific landscape in this area is a growing field of research.

The term map of the main analysis (Fig. 4) reveals five thematic clusters, with three major clusters. One major cluster (red, Fig. 4) is mostly concerned with cognitive functions in general in brain tumour patients. More specifically, the research addressed consequences of surgical resections on cognitive functions. Concerning modality of cognitive assessment, previous research focused on cognitive screenings (e.g. “montreal cognitive assessment” or “mini mental state examination”) in spite of evidence that those might lack the required sensitivity to detect impairment in brain tumour patients [29, 30] and the use in other clinical conditions is controversially discussed [31–34]. A second cluster (green, Fig. 4) represents a focus on cognitive functions in paediatric brain tumour patients, a population followed carefully and comprehensively with serial cognitive assessment and QoL measures by the paediatric haemato-oncological community. A third major cluster (blue, Fig. 4) comprises terms related to psychological factors in brain tumour patients (e.g. “fatigue”, “anxiety” or “depression”) and their influence on QoL [35–38]. Diagnosis and treatment of a brain tumour usually entail a significant psychological burden for both the patients and their caregivers [39–44]. Several studies observed that perceived deficits in cognitive functions more strongly correlate with self-reported anxiety, depression and mental fatigue than with objective cognitive test performance [45–49].

A first minor cluster (purple, Fig. 4) represents research on long-term survivors of a brain tumour (e.g. “primary central nervous system lymphoma” and “clinical trial”) as many patients under the age of 65 years can be cured with intensive chemotherapy regimens despite the extremely aggressive nature of primary central nervous system lymphomas [50–54]. A second minor cluster (yellow, Fig. 4) indicates the importance of social cognition and cognitive rehabilitation (e.g. “social skill”, “intervention” and “improvement”).

Until now, other components of sociocognitive functions, for instance Theory of Mind, empathy or social problem-solving, are missing in the term maps of the main analysis (Figs. 3, 4 and 5) as sociocognitive functions have only recently been considered in research on brain tumour patients and have therefore not yet reached the predefined thresholds in order to be registered in this broad automated literature analysis.

Previous research on cognitive outcome after surgery mainly focused on WHO-grade IV tumours with inconsistent results [7, 55–60]. To uncover the impact of (socio)cognitive functions in patients, who have to cope with potential impairments of those functions for many years or even decades, an additional subanalysis focusing on patient populations with prolonged manageable disease control reveals three major (red, blue, green) and one minor (yellow) thematic cluster (Fig. 7). The first major cluster represents the focus on consequences of surgical resections (red, Fig. 7). In this vein, a recent study on lower grade gliomas reported frequent cognitive decline after resective surgery on diffuse glioma [19]. In previous studies, a focus was placed on attention since attention is required for almost every practical activity and is therefore essential to higher-order cognitive functions. Serious attention problems often contribute to impaired recovery in other functional domains [61]. The blue cluster (Fig. 7) represents studies on patients with potentially curable brain tumours (e.g. “primary central nervous system lymphoma”) and one major outcome measure, health related QoL, is represented within the green cluster (Fig. 7). As for the main analysis, the subanalysis confirms that recent research also focuses on influences of psychological constructs (Fig. 7 yellow cluster), for instance “anxiety” and “depression” [35–38]. The subanalysis further confirms that sociocognitive functions are not represented in the term maps until now as those abilities have only recently been considered in research on brain tumour patients. This further highlights the necessity to include social cognition in future research on brain tumour patients because it encompasses relevant functions for occupational and social functioning and eventually for general QoL in these patients.

Concerning the development of the scientific landscape (Figs. 5 and 8), early research on cognitive functions in brain tumour patients focused on assessment of cognitive functions as a relevant outcome measure beside overall- and progression-free survival in clinical trials. Since both the treatment and the (residual) brain tumour itself might affect the individual’s ability to function in everyday life situations, “quality of survivorship” has become an additional research focus and survival alone is no longer considered an adequate single outcome measure [62] especially in patient populations that have to cope with potential impairments of (socio)cognitive functions for many years or even decades. In this vein, current research also focuses on interactions between cognitive functions and psychological constructs, for instance “anxiety” and “depression” since those constructs are important for general and mental health and therefore for “quality of survivorship”, for instance in terms of reintegration into social and occupational roles. For many individuals, occupational reintegration represents one of the most important indicators of being rehabilitated into a normal life after being ill [63, 64].

(Meta)data from all 664 documents were used to identify the most prolific authors and the most impactful sources (Figs. 9 and 10). The total number of authors associated with the research field is high, but only 91 individuals have been associated with five or more relevant publications. Furthermore, the largest set of related authors consists of 40 well interconnected individuals.

The map charting the 31 most relevant sources (Fig. 10) reveals that research on cognitive and sociocognitive functions in brain tumour patients has been published in general medical, neurological and neurosurgical journals but predominantly in journals focusing on Neuro-Oncology, for instance the “Journal of Neuro-Oncology” and “Neuro-Oncology”. Furthermore, journals focusing on paediatric brain tumour patients were represented. Another interesting but also smaller contribution comes from psycho-oncological and neuropsychological journals (e.g. “Psycho-Oncology” and “Neuropsychologia”), again highlighting the interaction of psychological and (neuro)cognitive functions in patients with brain tumours.

A bibliometric analysis with computational algorithms such as the one used in this paper can only provide an overview of trends in research and is limited by certain factors: The thematic analysis is based solely on the frequency of term occurrence and co-occurrence without further semantic evaluation of content. Including meeting abstracts into the textual analysis might also have distorted the map visualizations in favour of studies that were published first as abstracts and later on as full research article. Furthermore, the input data were extracted using elaborated search strings but might still have missed publications that have less specific titles.

Despite the limitations mentioned above, this bibliometric analysis of literature on cognitive and sociocognitive functions in patients with brain tumours provides an insightful overview of the development and structure of the scientific landscape and also highlights fields of research that should be considered in further studies.

## References

[1] Aaronson NK, Taphoorn MJB, Heimans JJ, Postma TJ, Gundy CM, Beute GN, Slotman BJ, Klein M (2011). Compromised health-related quality of life in patients with low-grade glioma. J Clin Oncol.

[2] Bunevicius A, Tamasauskas S, Deltuva VP, Tamasauskas A, Radziunas A, Bunevicius R (2014). Predictors of health-related quality of life in neurosurgical brain tumor patients: focus on patient-centered perspective. Acta Neurochir.

[3] Hahn CA, Dunn RH, Logue PE (2003). Prospective study of neuropsychologic testing and quality-of-life assessment of adults with primary malignant brain tumors. Int J Radiat Oncol Biol Phys.

[4] Scheibel RS, Meyers CA, Levin VA (1996). Cognitive dysfunction following surgery for intracerebral glioma: influence of histopathology, lesion location, and treatment. J Neuro-Oncol.

[5] Weitzner MA, Meyers CA, Byrne K (1996). Psychosocial functioning and quality of life in patients with primary brain tumors. J Neurosurg.

[6] Zucchella C, Bartolo M, Di Lorenzo C (2013). Cognitive impairment in primary brain tumors outpatients: a prospective cross-sectional survey. J Neuro Oncol.

[7] Habets EJJ, Kloet A, Walchenbach R, Vecht CJ, Klein M, Taphoorn MJ (2014). Tumour and surgery effects on cognitive functioning in high-grade glioma patients. Acta Neurochir.

[8] Schagen SB, Klein M, Reijneveld JC, Brain E, Deprez S, Joly F, Scherwath A, Schrauwen W, Wefel JS (2014). Monitoring and optimising cognitive function in cancer patients: present knowledge and future directions. EJC Suppl.

[9] Taphoorn MJB, Klein M (2004). Cognitive deficits in adult patients with brain tumours. Lancet Neurol.

[10] Correa DD (2010). Neurocognitive function in brain tumors. Curr Neurol Neurosci Rep.

[11] Douw L, Klein M, Fagel SSAA, van den Heuvel J, Taphoorn MJ, Aaronson NK, Postma TJ, Vandertop WP, Mooij JJ, Boerman RH, Beute GN, Sluimer JD, Slotman BJ, Reijneveld JC, Heimans JJ (2009). Cognitive and radiological effects of radiotherapy in patients with low-grade glioma: long-term follow-up. Lancet Neurol.

[12] Klein M (2012). Neurocognitive functioning in adult WHO grade II gliomas: impact of old and new treatment modalities. Neuro-Oncology.

[13] Adolphs R (2001). The neurobiology of social cognition. Curr Opin Neurobiol.

[14] Baron-Cohen S, Wheelwright S (2004). The empathy quotient: an investigation of adults with Asperger syndrome or high functioning autism, and normal sex differences. J Autism Dev Disord.

[15] Lovely MP, Stewart-Amidei C, Page M, Mogensen K, Arzbaecher J, Lupica K, Maher ME (2013). A new reality: long-term survivorship with a malignant brain tumor. Oncol Nurs Forum.

[16] Ownsworth T, Chambers SK, Hawkes A, Walker DG, Shum D (2011). Making sense of brain tumour: a qualitative investigation of personal and social processes of adjustment. Neuropsychol Rehabil.

[17] Payne S, Jarrett N, Jeffs D, Brown L (2001). Implications of social isolation during cancer treatment. The implications of residence away from home during cancer treatment on patients’ experiences: a comparative study. Health Place.

[18] Cobb S (1976). Social support as a moderator of life stress. Psychosom Med.

[19] Hendriks EJ, Habets EJJ, Taphoorn MJB, Douw L, Zwinderman AH, Vandertop WP, Barkhof F, Klein M, de Witt Hamer PC (2018). Linking late cognitive outcome with glioma surgery location using resection cavity maps. Hum Brain Mapp.

[20] Klein M, Taphoorn MJB, Heimans JJ, van der Ploeg H, Vandertop WP, Smit EF, Leenstra S, Tulleken CA, Boogerd W, Belderbos JS, Cleijne W, Aaronson NK (2001). Neurobehavioral status and health-related quality of life in newly diagnosed high-grade glioma patients. J Clin Oncol.

[21] Meyers CA, Brown PD (2006). Role and relevance of neurocognitive assessment in clinical trials of patients with CNS tumors. J Clin Oncol.

[22] Tucha O, Smely C, Preier M, Lange KW (2000). Cognitive deficits before treatment among patients with brain tumors. Neurosurgery.

[23] van Eck NJ, Waltman L (2011) Text mining and visualization using VOSviewer. arXiv preprint:1–5

[24] van Eck NJ, Waltman L (2010). Software survey: VOSviewer, a computer program for bibliometric mapping. Scientometrics.

[25] van Eck NJ, Waltman L, Noyons ECM, Buter RK (2010). Automatic term identification for bibliometric mapping. Scientometrics.

[26] van Eck NJ, Waltman L (2013) VOSviewer manual. Univeristeit Leiden, Leiden 1(1)

[27] Popkirov S, Jungilligens J, Schlegel U, Wellmer J (2018). Research on dissociative seizures: a bibliometric analysis and visualization of the scientific landscape. Epilepsy Behav.

[28] Bigler ED (2017). Structural neuroimaging in neuropsychology: history and contemporary applications. Neuropsychology.

[29] Meyers CA, Wefel JS (2003). The use of the mini-mental state examination to assess cognitive functioning in cancer trials: no ifs, ands, buts, or sensitivity. J Clin Oncol.

[30] Robinson GA, Biggs V, Walker DG (2015). Cognitive screening in brain tumors: short but sensitive enough?. Front Oncol.

[31] Kawada T (2019). Montreal Cognitive Assessment (MoCA) and its memory tasks for detecting mild cognitive impairment. Neurol Sci.

[32] Li X, Jia S, Zhou Z, Jin Y, Zhang X, Hou C, Zheng W, Rong P, Jiao J (2018). The role of the Montreal Cognitive Assessment (MoCA) and its memory tasks for detecting mild cognitive impairment. Neurol Sci.

[33] Milosevich E, Pendlebury S, Demeyere N (2019). Reply to: “Diagnostic test accuracy of the Montreal Cognitive Assessment in the detection of post-stroke cognitive impairment under different stages and cutoffs: a systematic review and meta-analysis”. Neurol Sci.

[34] Shi D, Chen X, Li Z (2018). Diagnostic test accuracy of the Montreal Cognitive Assessment in the detection of post-stroke cognitive impairment under different stages and cutoffs: a systematic review and meta-analysis. Neurol Sci.

[35] Arndt J, Das E, Schagen SB, Reid-Arndt SA, Cameron LD, Ahles TA (2014). Broadening the cancer and cognition landscape: the role of self-regulatory challenges. Psychooncology.

[36] Arnold SD, Forman LM, Brigidi BD, Carter KE, Schweitzer HA, Quinn HE, Guill AB, Herndon JE, Raynor RH (2008). Evaluation and characterization of generalized anxiety and depression in patients with primary brain tumors. Neuro-Oncology.

[37] Noll KR, Bradshaw ME, Weinberg JS, Wefel JS (2017). Relationships between neurocognitive functioning, mood, and quality of life in patients with temporal lobe glioma. Psychooncology.

[38] Richter A, Woernle CM, Krayenbühl N, Kollias S, Bellut D (2015). Affective symptoms and white matter changes in brain tumor patients. World Neurosurg.

[39] Boele FW, Heimans JJ, Aaronson NK, Taphoorn MJ, Postma TJ, Reijneveld JC, Klein M (2013). Health-related quality of life of significant others of patients with malignant CNS versus non-CNS tumors: a comparative study. J Neurooncol.

[40] Boele FW, Douw L, Reijneveld JC, Robben R, Taphoorn MJ, Aaronson NK, Heimans JJ, Klein M (2015). Health-related quality of life in stable, long-term survivors of low-grade glioma. J Clin Oncol.

[41] Boele FW, Rooney AG, Grant R, Klein M (2015). Psychiatric symptoms in glioma patients: from diagnosis to management. Neuropsychiatr Dis Treat.

[42] Boele FW, van Uden-Kraan CF, Hilverda K, Reijneveld JC, Cleijne W, Klein M, Verdonck-de Leeuw IM (2016). Attitudes and preferences toward monitoring symptoms, distress, and quality of life in glioma patients and their informal caregivers. Support Care Cancer.

[43] Francis SR, Hall EOC, Delmar C (2019) Ethical dilemmas experienced by spouses of a partner with brain tumour. Nurs Ethics:1–11. 10.1177/0969733019857790 10.1177/0969733019857790 31319743

[44] Ownsworth T, Goadby E, Chambers SK (2015). Support after brain tumor means different things: family caregivers’ experiences of support and relationship changes. Front Oncol.

[45] Costa DSJ, Fardell JE (2019). Why are objective and perceived cognitive function weakly correlated in patients with cancer?. J Clin Oncol.

[46] Cull A, Hay C, Love SB (1996). What do cancer patients mean when they complain of concentration and memory problems?. Br J Cancer.

[47] Strober LB, Binder A, Nikelshpur OM (2016). The perceived deficits questionnaire: perception, deficit, or distress?. Int J MS Care.

[48] Pranckeviciene A, Deltuva VP, Tamasauskas A, Bunevicius A (2017). Association between psychological distress, subjective cognitive complaints and objective neuropsychological functioning in brain tumor patients. Clin Neurol Neurosurg.

[49] Di Iulio F, Cravello L, Shofany J (2019). Neuropsychological disorders in non-central nervous system cancer: a review of objective cognitive impairment, depression, and related rehabilitation options. Neurol Sci.

[50] Gerstner ER, Batchelor TT (2010). Primary central nervous system lymphoma. Arch Neurol.

[51] Juergens A, Pels H, Rogowski S (2010). Long-term survival with favorable cognitive outcome after chemotherapy in primary central nervous system lymphoma. Ann Neurol.

[52] Korfel A, Schlegel U (2013). Diagnosis and treatment of primary CNS lymphoma. Nat Rev Neurol.

[53] Pels H, Schmidt-Wolf IGH, Glasmacher A, Schulz H, Engert A, Diehl V, Zellner A, Schackert G, Reichmann H, Kroschinsky F, Vogt-Schaden M, Egerer G, Bode U, Schaller C, Deckert M, Fimmers R, Helmstaedter C, Atasoy A, Klockgether T, Schlegel U (2003). Primary central nervous system lymphoma: results of a pilot and phase II study of systemic and intraventricular chemotherapy with deferred radiotherapy. J Clin Oncol.

[54] Seidel S, Korfel A, Kowalski T, Margold M, Ismail F, Schroers R, Baraniskin A, Pels H, Martus P, Schlegel U (2019). HDMTX-based induction therapy followed by consolidation with conventional systemic chemotherapy and intraventricular therapy (modified Bonn protocol) in primary CNS lymphoma: a monocentric retrospective analysis. Neurological Research and Practice.

[55] Dallabona M, Sarubbo S, Merler S, Corsini F, Pulcrano G, Rozzanigo U, Barbareschi M, Chioffi F (2017). Impact of mass effect, tumor location, age, and surgery on the cognitive outcome of patients with high-grade gliomas: a longitudinal study. Neurooncol Pract.

[56] Mandonnet E, de Witt Hamer PC, Poisson I (2015). Initial experience using awake surgery for glioma: oncological, functional, and employment outcomes in a consecutive series of 25 cases. Neurosurgery.

[57] Noll KR, Weinberg JS, Ziu M, Benveniste RJ, Suki D, Wefel JS (2015). Neurocognitive changes associated with surgical resection of left and right temporal lobe glioma. Neurosurgery.

[58] Satoer D, Visch-Brink E, Smits M, Kloet A, Looman C, Dirven C, Vincent A (2014). Long-term evaluation of cognition after glioma surgery in eloquent areas. J Neurooncol.

[59] Talacchi A, Santini B, Savazzi S, Gerosa M (2011). Cognitive effects of tumour and surgical treatment in glioma patients. J Neurooncol.

[60] Wu AS, Witgert ME, Lang FF, Xiao L, Bekele BN, Meyers CA, Ferson D, Wefel JS (2011). Neurocognitive function before and after surgery for insular gliomas. J Neurosurg.

[61] Bogdanova Y, Yee MK, Ho VT, Cicerone KD (2016). Computerized cognitive rehabilitation of attention and executive function in acquired brain injury: a systematic review. J Head Trauma Rehabil.

[62] Amidei C (2018). Symptom-based interventions to promote quality survivorship. Neuro Oncol.

[63] Alaszewski A, Alaszewski H, Potter J, Penhale B (2007). Working after a stroke: survivors’ experiences and perceptions of barriers to and facilitators of the return to paid employment. Disabil Rehabil.

[64] Saunders SL, Nedelec B (2014). What work means to people with work disability: a scoping review. J Occup Rehabil.

