# Correction to: Research on cognitive and sociocognitive functions in patients with brain tumours: a bibliometric analysis and visualization of the scientific landscape

**DOI:** 10.1007/s10072-021-05413-w

**Published:** 2021-07-09

**Authors:** Milena Pertz, Stoyan Popkirov, Uwe Schlegel, Patrizia Thoma

**Affiliations:** 1 Department of Neurology, University Hospital Knappschaftskrankenhaus, Ruhr-University Bochum, In der Schornau 23-25, D-44892 Bochum, Germany; 2 Neuropsychological Therapy Centre (NTC)/Clinical Neuropsychology, Faculty of Psychology, Ruhr-University Bochum, Universitätsstraße 150, D-44780 Bochum, Germany

**Correction to: Neurological Sciences (2020) 41:1437–1449**

**https://doi.org/10.1007/s10072-020-04276-x**

The article “Research on cognitive and sociocognitive functions in patients with brain tumours: a bibliometric analysis and visualization of the scientific landscape”, written by Milena Pertz, Stoyan Popkirov, Uwe Schlegel and Patrizia Thoma, was originally published Online First without Open Access. After publication in volume 41, issue 6, page 1437–1449 the author decided to opt for Open Choice and to make the article an Open Access publication. Therefore, the copyright of the article has been changed to © The Author(s) 2020 and the article is forthwith distributed under the terms of the Creative Commons Attribution 4.0 International License, which permits use, sharing, adaptation, distribution and reproduction in any medium or format, as long as you give appropriate credit to the original author(s) and the source, provide a link to the Creative Commons licence, and indicate if changes were made. The images or other third party material in this article are included in the article’s Creative Commons licence, unless indicated otherwise in a credit line to the material. If material is not included in the article’s Creative Commons licence and your intended use is not permitted by statutory regulation or exceeds the permitted use, you will need to obtain permission directly from the copyright holder. To view a copy of this licence, visit http://creativecommons.org/licenses/by/4.0.

The original article has been corrected.

**Open Access** This article is licensed under a Creative Commons Attribution 4.0 International License, which permits use, sharing, adaptation, distribution and reproduction in any medium or format, as long as you give appropriate credit to the original author(s) and the source, provide a link to the Creative Commons licence, and indicate if changes were made. The images or other third party material in this article are included in the article's Creative Commons licence, unless indicated otherwise in a credit line to the material. If material is not included in the article's Creative Commons licence and your intended use is not permitted by statutory regulation or exceeds the permitted use, you will need to obtain permission directly from the copyright holder. To view a copy of this licence, visit http://creativecommons.org/licenses/by/4.0/.

